# Complete Genome Sequence of Mycobacterium xenopi JCM15661^T^, Obtained Using Nanopore and Illumina Sequencing Technologies

**DOI:** 10.1128/MRA.01583-19

**Published:** 2020-03-05

**Authors:** Mitsunori Yoshida, Hanako Fukano, Takanori Asakura, Junzo Hisatsune, Yoshihiko Hoshino

**Affiliations:** a Department of Mycobacteriology, Leprosy Research Center, National Institute of Infectious Diseases, Tokyo, Japan; b Antimicrobial Resistance Research Center, National Institute of Infectious Diseases, Tokyo, Japan; University of Maryland School of Medicine

## Abstract

Mycobacterium xenopi is a slow-growing mycobacterial organism for which pathogenic features are unclear. Here, we report the complete genome sequence of an M. xenopi type strain. This sequence will provide essential information for future taxonomic and comparative genome studies of these mycobacteria.

## ANNOUNCEMENT

Mycobacterium xenopi is a slow-growing, scotochromogenic, thermophilic nontuberculous mycobacterial (NTM) species (1). It was originally isolated from skin lesions found on a toad (Xenopus laevis), and its infection of humans was confirmed in 1959 (2). In worldwide surveillance of NTM lung disease in 2013, M. xenopi was the third most frequently isolated species (3). Of note, isolation of this species was limited to some European countries and Canada, whereas it was seldom isolated in Asia, Australia, and South America (3). The standard treatment regimen remains to be determined, resulting in relatively poor prognoses among NTM lung diseases (4–8). Here, we report the complete genome sequence of an M. xenopi type strain, helping us to understand the pathogenic features.

M. xenopi strain JCM15661^T^ (i.e., ATCC 19250 or DSM43995) was purchased from the Japan Collection of Microorganisms. The strain was inoculated on 2% Ogawa medium and incubated at 37°C for 2 weeks. Genomic DNA was extracted by a standard phenol-chloroform method (9, 10). Long-read sequence reads (96,392 reads) were obtained with the MinION platform (Oxford Nanopore Technologies, Oxford, UK). Approximately 80 ng of genomic DNA was used for library preparation with the SQK-RAD004 rapid barcoding sequencing kit (Oxford Nanopore Technologies), in accordance with the manufacturer’s protocol. The library was loaded on a SpotON Mk I (R9.4) flow cell and sequenced using MinKNOW v.19.12.2. Raw sequence data (fast5 format) were base called using Guppy v.3.4.1 software. Short (<500-bp) and/or low-quality (quality scores of <10) reads were filtered using Filtlong software (https://github.com/rrwick/Filtlong). The remining reads (80,299 reads, with an average read length of 6,315 bp) were *de novo* assembled into one contig (4,917,655 bp) with the suggestCircular flag, using Canu v.1.8 (11) with the following parameters: CorOutCoverage, 200; ContigFilter, 5 10000 1.0 1.0 10; and genomeSize, 4.93M. The assembled genome was circularized by manually trimming the repeated sequences. Using the same genomic DNA sample as described above, Illumina paired-end (2 × 300-bp) reads (799,476 reads) were obtained with the MiSeq system (Illumina, San Diego, CA). A DNA library for sequencing of Illumina reads was prepared using the QIAseq FX DNA library kit (Qiagen). After quality was checked using FastQC v.0.11.5 (http://www.bioinformatics.babraham.ac.uk/projects/fastqc), these reads were mapped to the assembly with Burrows-Wheeler Aligner v.0.7.17 (12) for sequence and assembly error correction with Pilon v.1.2.3 (13). The resulting sequence was annotated using the DFAST v.1.1.15 pipeline (14), and the average nucleotide identity (ANI) was calculated using JSpeciesWS v.3.3.0 (15), with default settings.

The chromosome of M. xenopi JCM15661^T^ is 4,917,655 bp (G+C content, 65.9%). The ANI values with respect to two reported draft genomes of the strain (named DSM43995 and NCTC10042 in the NCBI database) were 99.89% (versus strain DSM43995) and 99.87% (versus strain NCTC10042). Also, the ANI values with respect to draft genomes of Mycobacterium heckeshornense (strain RLE) and Mycobacterium noviomagense (strain DSM45145), which are the mycobacterial species phylogenetically closest to M. xenopi (16–18), were 89.43% and 82.93%, respectively, confirming the taxonomic position of M. xenopi. The numbers of predicted coding sequences, rRNA operons, and tRNAs in the genome were 4,898, 6, and 47, respectively, nearly equivalent to those of the two previously reported draft genomes of M. xenopi. The complete genome sequence of M. xenopi JCM15661^T^ provides essential data for future taxonomic and comparative genome studies.

### Data availability.

The genome sequence and annotations of M. xenopi were deposited in DDBJ/EMBL/GenBank under accession number AP022314. Raw sequence data for strain JCM15661^T^ were deposited under DRA accession numbers DRR201556 (MinION) and DRR201557 (Illumina).
